# A novel Hellinger distance-based regret theory method for spherical fuzzy decision making model and its application in logistics

**DOI:** 10.1038/s41598-024-63876-1

**Published:** 2024-06-17

**Authors:** Haiping Ren, Hui Zhou

**Affiliations:** 1 Teaching Department of Basic Subjects, Jiangxi University of Science and Technology, Nanchang, 330013 China; 2 School of Mathematics and Computer Science, Yichun University, Yichun, 336000 China

**Keywords:** Hellinger distance, Spherical fuzzy set, Regret theory, Multi-attribute decision making, Engineering, Mathematics and computing

## Abstract

Actual decision making problems are often based on the company decision maker’s behavior factors, such as risk attitude, subjective preference, etc. Regret theory can well express the behavior of the decision maker. In this pursuit, a novel decision making method was developed, based on the regret theory for the multi-attribute decision making problem, in which attribute values were expressed by spherical fuzzy numbers. Distance measurement not only has extensive applications in fields such as pattern recognition and image processing, but also plays an important role in the research of fuzzy decision theory. The existing distance measures of spherical fuzzy set either have special cases of anti-intuition or are more complex in calculation, so finding suitable distance measures is also an important research topic in the decision-making theory of spherical fuzzy set. For this reason, we first establish a new distance of spherical fuzzy sets based on Hellinger distance of probability distribution. A decision maker’s perception utility value function is proposed using the new distance formula, which is used to measure the regretful and rejoice value. Then we establish an optimization model for solving the attribute weights, when the information of attribute weight was partially known. Subsequently, the comprehensive perceived utility values were utilized to rank the order of the alternatives. Finally, a numerical example of assessment of logistics providers is used to show that the new decision making method is effective and feasible.

## Introduction

The concept of fuzzy sets, firstly founded by Zadeh in 1965, due to the need to describe and process the large number of fuzzy phenomena that exist in the real world. In people’s thinking, many concepts are fuzzy, such as young, very large, warm, evening, etc.^1^. The attributes of objects described by these concepts cannot be simply answered with “yes” or “no”. Therefore, the concept of fuzzy sets emerged in order to describe and deal with these fuzzy concepts^2^. By establishing appropriate membership functions and utilizing relevant operations and transformations of fuzzy sets, fuzzy objects can be analyzed. This method provides an effective mathematical tool for describing and dealing with fuzzy phenomena. It has become a powerful tool to describe the human brain’s thinking in processing fuzzy information and it has many successful applications in dealing with various practical problems, such as automatic control, pattern recognition, and medical diagnosis wherein some vague or uncertain characteristics exist^3–8^.

**Figure 1:**
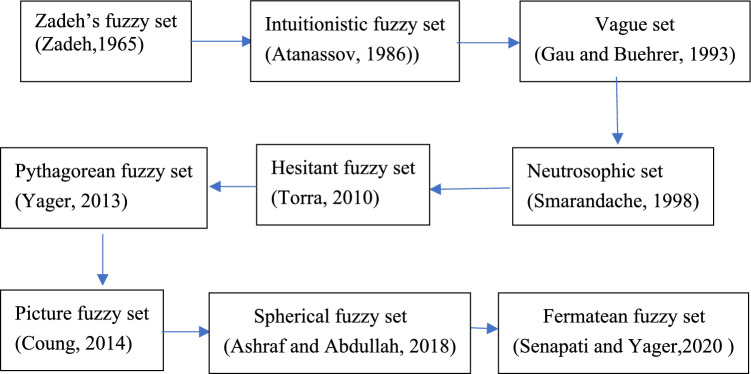
The extensions of fuzzy sets.

Although Zadeh’s fuzzy set theory can describe and handle fuzziness, it still has some limitations. It mainly relies on membership functions to describe the degree of an element belonging to a set. However, in many cases, it is difficult to fully characterize fuzziness using only membership functions^9, 10^. For example, when dealing with certain complex problems, we not only need to know the degree of an element belonging to a set, but also need to understand the degree of the element not belonging to the set or the hesitation degree of belonging to the set. Therefore, to more comprehensively describe and handle fuzziness, more parameters need to be introduced in the concepts of extensions of Zadeh’s fuzzy sets. In recent years, some extensions of Zadeh’s fuzzy set were proposed, they are intuitionistic fuzzy set, vague set, picture fuzzy set, neutrosophic set etc. Some extensions of Zadeh’s fuzzy set and applications can be found in Table 1. Figure 1 illustrates the extensions of fuzzy sets.

**Table 1:** Some extensions of Zadeh’s fuzzy set and applications.

References	Extensions of Zadeh’s fuzzy set	Applications
^11^	Intuitionistic fuzzy sets	Decision-making, pattern recognition, clustering problems
^12^	Vague set	Synergy development of regional industrial technology
^13, 14^	Picture fuzzy set	Food enterprise quality credit evaluation; Industrial Fund Selection
^15^	Fermatean fuzzy set	Decision-making
^16^	Neutrosophic set	Multi-class image segmentation
^17^	Spherical fuzzy sets	multiattribute group decision-making

Spherical fuzzy (SF) set is a newly extension of Zadeh’s fuzzy set, which was introduced by Ashraf and Abdullah^17^. A SF set can better describe abstain degree and refusal degree of the judgment than a fuzzy set or intuitionistic fuzzy set because SF set contains three parameters: membership degree and non-membership degree and refusal degree. SF sets have been proved as an effective tool in expressing the uncertainty or fuzzy information in actual applications^18–21^.

Multi-attribute/multi-criteria decision making (MADM/MACD) models have extensive applications in many fields, including engineering, technology, economy, management, military, and so on^22, 23^. But most of MADM methods do not consider the influence of behavior of the decision-maker (DM) in the decision process, because of the assumption of the complete rationality of DM. However, the actual evaluation process often accompanies the behavior factors of the DM. Taking into account DM’s bounded rationality in multi-attribute decision-making (MADM) processes is more realistic. Therefore, MADM methods based on behavioral decision theory have received widespread attention and research in the past decade. In recent years, prospect theory and regret theory have attracted widely attention and based on these theories some decision making methods have been put forward^24–27^. Pen and Dai^28^ suggested that the regret theory has many advantages over the prospect theory in the actual decision process. For example, in decision making, if regret theory is applied, DMs need not give reference points, and the decision function involves fewer parameters in the calculation formula, which makes the calculation simpler^29, 30^. One task of this article is to develop a new evaluation method based on SF information.

Fourth-party logistics (4PLs) refer to a comprehensive logistics service model that includes not only traditional third-party logistics (3PLs) services but also higher-level supply chain management and coordination services^31^. 4PL providers typically have higher technical capabilities and management levels, enabling them to achieve visualized and intelligent management of the supply chain through information technology means, thereby improving logistics efficiency and reducing costs^32^. Logistics provider evaluation is an important part of selecting the right logistics provider for a business^33^. Evaluating logistics providers can help businesses understand their capabilities and performance, thereby better controlling logistics costs, improving logistics efficiency, reducing risks, etc.^34, 35^. Evaluating logistics providers is of great significance for businesses to select the right logistics providers, reduce procurement risks, improve logistics efficiency, control logistics costs, and promote supply chain collaboration^36^. Due to the presence of many difficult-to-quantify qualitative indicators in the evaluation of logistics providers, the use of fuzzy numbers or linguistic terms to express these indicators is more accurate. Guo et al.^37^ introduces a improved TODIM method for evaluation of emergency logistics suppliers based on probabilistic linguistic term sets. Jovčić et al.^38^ developed the fuzzy AHP method and TOPSIS method on the evaluation of emergency logistics suppliers based on linguistic terms and triangular fuzzy numbers. Aydn et al.^39^ put forward a novel neutrosophic MCDM method for the evaluation of fourth party logistics firms.There is still very little research on the use of SF sets for the assessment of logistics providers. So far, no one has extended the regret theory to SF decision making methods. In this pursuit, the present study developed a new regret theory based MADM method in solving the assessment of logistics providers, wherein the attribute values were expressed by SF numbers.

The organization of this manuscript is as follows: Section “Preliminary knowledge” introduces the concepts of SF numbers and puts forward a new spherical fuzzy distance. Section “New spherical fuzzy MADM method based on Helinger distance and regret theory” introduces the relevant knowledge of regret theory and develops a new MADM method based on the regret theory. Section “Application of assessment of logistics provider” provides two examples to illustrate the effectiveness and feasibility of the new proposed MADM method. Finally, Section “Conclusions” presents the conclusions of this study.

## Preliminary knowledge

Some concepts about SF sets will be first recalled, and then we will establish a new distance formula based on Hillinger distance and study the properties of the new distance measure.

### Spherical fuzzy set

#### Definition 1

Let $\mathscr{T}$ be a given domain. Gündoğdu and Kahraman^18^ introduced the concept of SF as follows:

$$ \mathscr{M} = \{ \langle \Delta ,\sigma_{\mathscr{M}} (\Delta ),\varsigma_{\mathscr{M}} (\Delta ),\tau_{\mathscr{M}} (\Delta )\rangle \left| {\Delta \in \mathscr{T}} \right.\} . $$

Here, $\sigma_{\mathscr{M}} (\Delta ):\Delta \to [0,1]$, $\varsigma_{\mathscr{M}} (\Delta ):\Delta \to [0,1]$, and $\tau_{\mathscr{M}} (\Delta ):\Delta \to [0,1]$ represent the membership degree, neutrality, and non-membership degree of elements $\Delta$ belonging to $\mathscr{M}$ in $\mathscr{T}$, respectively, and for all $\Delta \in \mathscr{T}$, $0 \le \sigma_{{_{\mathscr{M}} }}^{2} (\Delta ) + \varsigma_{{_{\mathscr{M}} }}^{2} (\Delta ) + \tau_{{_{\mathscr{M}} }}^{2} (\Delta ) \le 1$. For each fuzzy subset in $\mathscr{T}$, $\pi_{\mathscr{M}} (\Delta ) = 1 - \sqrt {\sigma_{\mathscr{M}}^{2} (\varvec{t}) + \varsigma_{\mathscr{M}}^{2} (\varvec{t}) + \tau_{\mathscr{M}}^{2} (\varvec{t})}$ is called the rejection degree of element $\Delta$ belonging to $\mathscr{M}$ in $\mathscr{T}$. When $\mathscr{T}$ has only one element, $\mathscr{M} = < \mu_{\mathscr{M}} ,\eta_{\mathscr{M}} ,\upsilon_{\mathscr{M}} >$ is commonly referred to as a SF number.

#### Definition 2

Let $\mathscr{T} = \{ \Delta_{1} ,\Delta_{2} ,...,\Delta_{\varvec{n}} \}$ be a given domain. There are two SF sets $\mathscr{M} = \{ \langle \Delta ,\sigma_{\mathscr{M}} (\Delta ),\varsigma_{\mathscr{M}} (\Delta ),\tau_{\mathscr{M}} (\Delta )\rangle \left| {\Delta \in \mathscr{T}} \right.\}$ and $\mathscr{N} = \{ \langle \Delta ,\sigma_{\mathscr{N}} (\Delta ),\varsigma_{\mathscr{N}} (\Delta ),\tau_{\mathscr{N}} (\Delta )\rangle \left| {\Delta \in \mathscr{T}} \right.\}$ defined in $\mathscr{T}$. The operational laws are defined as follows (Gündoğdu and Kahraman^18^):

i. (i) $$\mathscr{M} \subseteq \mathscr{N} \Leftrightarrow \forall \Delta \in \mathscr{T},\sigma_{\mathscr{M}} (\Delta ) \le \sigma_{\mathscr{N}} (\Delta ),\varsigma_{\mathscr{M}} (\Delta ) \le \varsigma_{\mathscr{N}} (\Delta ),\tau_{\mathscr{M}} (\Delta ) \ge \tau_{\mathscr{N}} (\Delta )$$_;_
ii. $$\mathscr{M} = \mathscr{N} \Leftrightarrow \mathscr{M} \subseteq \mathscr{N} \wedge \mathscr{N} \subseteq \mathscr{M}$$
iii. $$\mathscr{M}^{c} = \{ \langle \Delta ,\sigma_{\mathscr{M}} (\Delta ),\varsigma_{\mathscr{M}} (\Delta ),\tau_{\mathscr{M}} (\Delta )\rangle \left| {\Delta \in \mathscr{T}} \right.\}$$

#### Definition 3

Let $\mathscr{S}\mathscr{F}\mathscr{S}(\mathscr{T})$ be the set of all SF sets on the domain $\mathscr{T}$. A real-valued function $\mathscr{D}:\mathscr{S}\mathscr{F}\mathscr{S}(\mathscr{T}) \times \mathscr{S}\mathscr{F}\mathscr{S}(\mathscr{T}) \to R$ is called a distance measure on $\mathscr{S}\mathscr{F}\mathscr{S}(\mathscr{T})$. If for any $\mathscr{M},\mathscr{N},\mathscr{O} \in \mathscr{S}\mathscr{F}\mathscr{S}(\mathscr{T})$, the function $\mathscr{D}$ satisfies the following properties (Ali and Garg^40^):

i. $$\mathscr{D}(\mathscr{M},\mathscr{N}) \ge 0$$
ii. $\mathscr{M} = \mathscr{N}$ if and only if $\mathscr{D}(\mathscr{M},\mathscr{N}) = 0$;
iii. $$\mathscr{D}\,(\mathscr{M},\mathscr{N}) = \mathscr{D}(\mathscr{N},\mathscr{M})$$;
iv. $$\mathscr{D}(\mathscr{M},\mathscr{N}) \le \mathscr{D}(\mathscr{M},\mathscr{O}) + \mathscr{D}(\mathscr{O},\mathscr{N})$$.

### A new spherical fuzzy Hellinger distance

#### Definition 4

Let $P = (p_{1} ,p_{2} , \cdots ,p_{n} )$ and $Q = (q_{1} ,q_{2} , \cdots ,q_{n} )$ be two discrete probability distributions. The Hillinger distance between these two probability distributions is defined as (Sengar et al.^41^):

1 $$ d_{H} (P,Q) = \sqrt {\frac{1}{2}\sum\limits_{i = 1}^{n} {(\sqrt {p_{i} } - \sqrt {q_{i} } )^{2} } } $$

Since its proposal, the Hillinger distance has been widely applied in fields such as data mining and cryptography^42–48^.

According to the Cauchy–Schwarz inequality, we can easily obtain the conclusion of Lemma 1.

#### Lemma 1

Let $R$ be the set of real numbers and $\alpha_{\varvec{i}} ,\beta_{\varvec{i}} ,\gamma_{\varvec{i}} \in R\,(\varvec{i} = 1,2,...,\varvec{n})$, then.

2 $$ \sqrt {\sum\limits_{{\varvec{i} = 1}}^{\varvec{n}} {(\alpha_{\varvec{i}} + \beta_{\varvec{i}} + \gamma_{\varvec{i}} )^{2} } } \le \sqrt {\sum\limits_{{\varvec{i} = 1}}^{\varvec{n}} {\alpha_{\varvec{i}}^{2} } } + \sqrt {\sum\limits_{{\varvec{i} = 1}}^{\varvec{n}} {\beta_{\varvec{i}}^{2} } } + \sqrt {\sum\limits_{{\varvec{i} = 1}}^{\varvec{n}} {\gamma_{\varvec{i}}^{2} } } . $$

#### Lemma 2

For given non-negative real numbers $\alpha_{k} ,\beta_{k} (k = 1,2,3)$, the following expression is always true:

3 $$ \sqrt {\alpha_{1} \beta_{1} } + \sqrt {\alpha_{2} \beta_{2} } + \sqrt {\alpha_{3} \beta_{3} } \le \sqrt {\alpha_{1} + \alpha_{2} + \alpha_{3} } \times \sqrt {\beta_{1} + \beta_{2} + \beta_{3} } . $$

Next, we will propose the Hillinger distance formula between two any SF sets based on Eq. (1).

#### Theorem 1

Let $\mathscr{T} = \{ \Delta_{1} ,\Delta_{2} ,...,\Delta_{\varvec{n}} \}$ be a given domain. There are two SF sets $\mathscr{M} = \{ \langle \Delta ,\sigma _{\mathscr{M}} (\Delta ),\varsigma _{\mathscr{M}} (\Delta ),\tau _{\mathscr{M}} (\Delta )\rangle \left| {\Delta  \in \mathscr{T}} \right.\}$ and $\mathscr{N} = \{ \langle \Delta ,\sigma _{ \mathscr{N}} (\Delta ),\varsigma _{ \mathscr{N}} (\Delta ),\tau _{ \mathscr{N}} (\Delta )\rangle \left| {\Delta  \in  \mathscr{T}} \right.\}$ defined in $\mathscr{T}$. Based on the Hillinger distance (1), a new information measurement between $\mathscr{M}$ and $\mathscr{N}$ is defined as follows:

4 $$ \mathscr{D}_{H} (\mathscr{M},\mathscr{N}) = \sqrt {\frac{1}{{{3}\varvec{n}}}\sum\limits_{{\varvec{i} = 1}}^{\varvec{n}} {\left( {\sqrt {\sigma_{\mathscr{M}} (\Delta_{\varvec{i}} )} - \sqrt {\sigma_{\mathscr{N}} (\Delta_{\varvec{i}} )} } \right)^{2} + \left( {\sqrt {\varsigma_{\mathscr{M}} (\Delta_{\varvec{i}} )} - \sqrt {\varsigma_{\mathscr{N}} (\Delta_{\varvec{i}} )} } \right)^{2} + \left( {\sqrt {\tau_{\mathscr{M}} (\Delta_{\varvec{i}} )} - \sqrt {\tau_{\mathscr{N}} (\Delta_{\varvec{i}} )} } \right)^{2} } } $$

Then $\mathscr{D}_{H} (\mathscr{M},\mathscr{N})$ is a distance measure. That is it satisfies (i)–(iv) of Definition 1.

#### Proof of Theorem 1

The property (i) is obviously true.

(ii) If $\mathscr{M} = \mathscr{N}$, then

5 $$ \forall \varvec{t} \in \mathscr{T},\mu_{\mathscr{M}} (\varvec{t}) = \mu_{\mathscr{N}} (\varvec{t}),\eta_{\mathscr{M}} (\varvec{t}) = \eta_{\mathscr{N}} (\varvec{t}),\upsilon_{\mathscr{M}} (\varvec{t}) = \upsilon_{\mathscr{N}} (\varvec{t}), $$

Therefore, it is obvious that $\mathscr{D}_{H} (\mathscr{M},\mathscr{N}) = 0$.

Conversely, If $$\mathscr{D}_{H} (\mathscr{M},\mathscr{N}) = 0$$, i.e.

6 $$ \mathscr{D}_{H} (\mathscr{M},\mathscr{N}) = \sqrt {\frac{1}{{{3}\varvec{n}}}\sum\limits_{{\varvec{i} = 1}}^{\varvec{n}} {\left( {\sqrt {\sigma_{\mathscr{M}} (\Delta_{\varvec{i}} )} - \sqrt {\sigma_{\mathscr{N}} (\Delta_{\varvec{i}} )} } \right)^{2} + \left( {\sqrt {\varsigma_{\mathscr{M}} (\Delta_{\varvec{i}} )} - \sqrt {\varsigma_{\mathscr{N}} (\Delta_{\varvec{i}} )} } \right)^{2} + \left( {\sqrt {\tau_{\mathscr{M}} (\Delta_{\varvec{i}} )} - \sqrt {\tau_{\mathscr{N}} (\Delta_{\varvec{i}} )} } \right)^{2} } } = 0, $$

Then $$\forall \Delta \in \mathscr{T},\sigma_{\mathscr{M}} (\Delta ) = \sigma_{\mathscr{N}} (\Delta ),\varsigma_{\mathscr{M}} (\Delta ) = \varsigma_{\mathscr{N}} (\Delta ),\tau_{\mathscr{M}} (\Delta ) = \tau_{\mathscr{N}} (\Delta )$$.

That is $\mathscr{M} = \mathscr{N}$_._ Thus, (ii) is proved.

(iii) It is obvious that

7 $$ \begin{aligned} \mathscr{D}_{H} (\mathscr{N},\mathscr{M}) & = \sqrt {\frac{1}{{{3}\varvec{n}}}\sum\limits_{{\varvec{i} = 1}}^{\varvec{n}} {\left[ {\left( {\sqrt {\sigma_{\mathscr{N}} (\Delta_{\varvec{i}} )} - \sqrt {\sigma_{\mathscr{M}} (\Delta_{\varvec{i}} )} } \right)^{2} + \left( {\sqrt {\varsigma_{\mathscr{N}} (\Delta_{\varvec{i}} )} - \sqrt {\varsigma_{\mathscr{M}} (\Delta_{\varvec{i}} )} } \right)^{2} + \left( {\sqrt {\tau_{\mathscr{N}} (\Delta_{\varvec{i}} )} - \sqrt {\tau_{\mathscr{M}} (\Delta_{\varvec{i}} )} } \right)^{2} } \right]} } \\ & = \sqrt {\frac{1}{{{3}\varvec{n}}}\sum\limits_{{\varvec{i} = 1}}^{\varvec{n}} {\left[ {\left( {\sqrt {\sigma_{\mathscr{M}} (\Delta_{\varvec{i}} )} - \sqrt {\sigma_{\mathscr{N}} (\Delta_{\varvec{i}} )} } \right)^{2} + \left( {\sqrt {\varsigma_{\mathscr{M}} (\Delta_{\varvec{i}} )} - \sqrt {\varsigma_{\mathscr{N}} (\Delta_{\varvec{i}} )} } \right)^{2} + \left( {\sqrt {\tau_{\mathscr{M}} (\Delta_{\varvec{i}} )} - \sqrt {\tau_{\mathscr{N}} (\Delta_{\varvec{i}} )} } \right)^{2} } \right]} } \\ &  = \mathscr{D}_{H} (\mathscr{M},\mathscr{N}). \\ \end{aligned} $$

Next, we prove (iv).

8 $$ \begin{aligned} & \left( {\sqrt {\sigma_{\mathscr{M}} (\Delta_{i} )} - \sqrt {\sigma_{\mathscr{N}} (\Delta_{i} )} } \right)^{2} \\ & = \left( {\sqrt {\sigma_{\mathscr{M}} (\Delta_{i} )} - \sqrt {\sigma_{\mathscr{O}} (\Delta_{i} )} + \sqrt {\sigma_{\mathscr{O}} (\Delta_{i} )} - \sqrt {\sigma_{\mathscr{N}} (\Delta_{i} )} } \right)^{2} \\ & = \left( {\sqrt {\sigma_{\mathscr{M}} (\Delta_{i} )} - \sqrt {\sigma_{\mathscr{O}} (\Delta_{i} )} } \right)^{2} + \left( {\sqrt {\sigma_{\mathscr{O}} (\Delta_{i} )} - \sqrt {\sigma_{\mathscr{N}} (\Delta_{i} )} } \right)^{2} \\ & \quad + 2\left( {\sqrt {\sigma_{\mathscr{M}} (\Delta_{i} )} - \sqrt {\sigma_{\mathscr{N}} (\Delta_{i} )} } \right)\left( {\sqrt {\sigma_{\mathscr{N}} (\Delta_{i} )} - \sqrt {\sigma_{\mathscr{O}} (\Delta_{i} )} } \right), \\ \end{aligned} $$

9 $$ \begin{aligned} & \left( {\sqrt {\varsigma_{\mathscr{M}} (\Delta_{i} )} - \sqrt {\varsigma_{\mathscr{N}} (\Delta_{i} )} } \right)^{2} \\ & = \left( {\sqrt {\varsigma_{\mathscr{M}} (\Delta_{i} )} - \sqrt {\varsigma_{\mathscr{O}} (\Delta_{i} )} + \sqrt {\varsigma_{\mathscr{O}} (\Delta_{i} )} - \sqrt {\varsigma_{\mathscr{N}} (\Delta_{i} )} } \right)^{2} \\ & = \left( {\sqrt {\varsigma_{\mathscr{M}} (\Delta_{i} )} - \sqrt {\varsigma_{\mathscr{O}} (\Delta_{i} )} } \right)^{2} + \left( {\sqrt {\varsigma_{\mathscr{O}} (\Delta_{i} )} - \sqrt {\varsigma_{\mathscr{N}} (\Delta_{i} )} } \right)^{2} \\ & \quad + 2\left( {\sqrt {\varsigma_{\mathscr{M}} (\Delta_{i} )} - \sqrt {\varsigma_{\mathscr{N}} (\Delta_{i} )} } \right)\left( {\sqrt {\varsigma_{\mathscr{N}} (\Delta_{i} )} - \sqrt {\varsigma_{\mathscr{O}} (\Delta_{i} )} } \right), \\ \end{aligned} $$

10 $$ \begin{aligned} & \left( {\sqrt {\tau_{\mathscr{M}} (\Delta_{i} )} - \sqrt {\tau_{\mathscr{N}} (\Delta_{i} )} } \right)^{2} \\ & = \left( {\sqrt {\tau_{\mathscr{M}} (\Delta_{i} )} - \sqrt {\tau_{\mathscr{O}} (\Delta_{i} )} + \sqrt {\tau_{\mathscr{O}} (\Delta_{i} )} - \sqrt {\tau_{\mathscr{N}} (\Delta_{i} )} } \right)^{2} \\ & = \left( {\sqrt {\tau_{\mathscr{M}} (\Delta_{i} )} - \sqrt {\tau_{\mathscr{O}} (\Delta_{i} )} } \right)^{2} + \left( {\sqrt {\tau_{\mathscr{O}} (\Delta_{i} )} - \sqrt {\tau_{\mathscr{N}} (\Delta_{i} )} } \right)^{2} \\ & \quad + 2\left( {\sqrt {\tau_{\mathscr{M}} (\Delta_{i} )} - \sqrt {\tau_{\mathscr{N}} (\Delta_{i} )} } \right)\left( {\sqrt {\tau_{\mathscr{N}} (\Delta_{i} )} - \sqrt {\tau_{\mathscr{O}} (\Delta_{i} )} } \right). \\ \end{aligned} $$

According to the Cauchy–Schwarz inequality to Eqs. (8)–(10), we have

11 $$ \begin{aligned} & \sum\limits_{{\varvec{i} = 1}}^{\varvec{n}} {\left( {\sqrt {\sigma_{\mathscr{M}} (\Delta_{i} )} - \sqrt {\sigma_{\mathscr{O}} (\Delta_{i} )} } \right)\left( {\sqrt {\sigma_{\mathscr{O}} (\Delta_{i} )} - \sqrt {\sigma_{\mathscr{N}} (\Delta_{i} )} } \right)} \\ & \le \sqrt {\sum\limits_{{\varvec{i} = 1}}^{\varvec{n}} {\left( {\sqrt {\sigma_{\mathscr{M}} (\Delta_{i} )} - \sqrt {\sigma_{\mathscr{O}} (\Delta_{i} )} } \right)^{2} \times \sum\limits_{{\varvec{i} = 1}}^{\varvec{n}} {\left( {\sqrt {\sigma_{\mathscr{O}} (\Delta_{i} )} - \sqrt {\sigma_{\mathscr{N}} (\Delta_{i} )} } \right)^{2} } } } , \\ \end{aligned} $$

12 $$ \begin{aligned} & \sum\limits_{{\varvec{i} = 1}}^{\varvec{n}} {\left( {\sqrt {\varsigma_{\mathscr{M}} (\Delta_{i} )} - \sqrt {\varsigma_{\mathscr{O}} (\Delta_{i} )} } \right)\left( {\sqrt {\varsigma_{\mathscr{O}} (\Delta_{i} )} - \sqrt {\varsigma_{\mathscr{N}} (\Delta_{i} )} } \right)} \\ & \le \sqrt {\sum\limits_{{\varvec{i} = 1}}^{\varvec{n}} {\left( {\sqrt {\varsigma_{\mathscr{M}} (\Delta_{i} )} - \sqrt {\varsigma_{\mathscr{O}} (\Delta_{i} )} } \right)^{2} \times \sum\limits_{{\varvec{i} = 1}}^{\varvec{n}} {\left( {\sqrt {\varsigma_{\mathscr{O}} (\Delta_{i} )} - \sqrt {\varsigma_{\mathscr{N}} (\Delta_{i} )} } \right)^{2} } } } , \\ \end{aligned} $$

13 $$ \begin{aligned} & \sum\limits_{{\varvec{i} = 1}}^{\varvec{n}} {\left( {\sqrt {\tau_{\mathscr{M}} (\Delta_{i} )} - \sqrt {\tau_{\mathscr{O}} (\Delta_{i} )} } \right)\left( {\sqrt {\tau_{\mathscr{O}} (\Delta_{i} )} - \sqrt {\tau_{\mathscr{N}} (\Delta_{i} )} } \right)} \\ & \le \sqrt {\sum\limits_{{\varvec{i} = 1}}^{\varvec{n}} {\left( {\sqrt {\tau_{\mathscr{M}} (\Delta_{i} )} - \sqrt {\tau_{\mathscr{O}} (\Delta_{i} )} } \right)^{2} \times \sum\limits_{{\varvec{i} = 1}}^{\varvec{n}} {\left( {\sqrt {\tau_{\mathscr{O}} (\Delta_{i} )} - \sqrt {\tau_{\mathscr{N}} (\Delta_{i} )} } \right)^{2} } } } . \\ \end{aligned} $$

Due to

14 $$ \mathscr{D}_{H} (\mathscr{M},\mathscr{O}) = \sqrt {\frac{1}{{{3}\varvec{n}}}\sum\limits_{{\varvec{i} = 1}}^{\varvec{n}} {\left[ {\left( {\sqrt {\sigma_{\mathscr{M}} (\Delta_{\varvec{i}} )} - \sqrt {\sigma_{\mathscr{O}} (\Delta_{\varvec{i}} )} } \right)^{2} + \left( {\sqrt {\varsigma_{\mathscr{M}} (\Delta_{\varvec{i}} )} - \sqrt {\varsigma_{\mathscr{O}} (\Delta_{\varvec{i}} )} } \right)^{2} + \left( {\sqrt {\tau_{\mathscr{M}} (\Delta_{\varvec{i}} )} - \sqrt {\tau_{\mathscr{O}} (\Delta_{\varvec{i}} )} } \right)^{2} } \right]} } , $$

15 $$ \mathscr{D}_{H} (\mathscr{N},\mathscr{O}) = \sqrt {\frac{1}{{{3}\varvec{n}}}\sum\limits_{{\varvec{i} = 1}}^{\varvec{n}} {\left[ {\left( {\sqrt {\sigma_{\mathscr{N}} (\Delta_{\varvec{i}} )} - \sqrt {\sigma_{\mathscr{O}} (\Delta_{\varvec{i}} )} } \right)^{2} + \left( {\sqrt {\varsigma_{\mathscr{N}} (\Delta_{\varvec{i}} )} - \sqrt {\varsigma_{\mathscr{O}} (\Delta_{\varvec{i}} )} } \right)^{2} + \left( {\sqrt {\tau_{\mathscr{N}} (\Delta_{\varvec{i}} )} - \sqrt {\tau_{\mathscr{O}} (\Delta_{\varvec{i}} )} } \right)^{2} } \right]} } . $$

According to inequalities (11)–(13) and Eqs. (14) and (15), we have

$$ \begin{aligned} & \mathscr{D}_{H}^{2} (\mathscr{M},\mathscr{N}) \\ & = \frac{1}{{{3}\varvec{n}}}\sum\limits_{{\varvec{i} = 1}}^{\varvec{n}} {\left[ {\left( {\sqrt {\sigma_{\mathscr{M}} (\Delta_{\varvec{i}} )} - \sqrt {\sigma_{\mathscr{N}} (\Delta_{\varvec{i}} )} } \right)^{2} + \left( {\sqrt {\varsigma_{\mathscr{M}} (\Delta_{\varvec{i}} )} - \sqrt {\varsigma_{\mathscr{N}} (\Delta_{\varvec{i}} )} } \right)^{2} + \left( {\sqrt {\tau_{\mathscr{M}} (\Delta_{\varvec{i}} )} - \sqrt {\tau_{\mathscr{N}} (\Delta_{\varvec{i}} )} } \right)^{2} } \right]} \\ & \le \frac{1}{{{3}\varvec{n}}}\sum\limits_{{\varvec{i} = 1}}^{\varvec{n}} {\left[ \begin{gathered} \left( {\sqrt {\sigma_{\mathscr{M}} (\Delta_{i} )} - \sqrt {\sigma_{\mathscr{O}} (\Delta_{i} )} } \right)^{2} + \left( {\sqrt {\sigma_{\mathscr{O}} (\Delta_{i} )} - \sqrt {\sigma_{\mathscr{N}} (\Delta_{i} )} } \right)^{2} + \left( {\sqrt {\varsigma_{\mathscr{M}} (\Delta_{i} )} - \sqrt {\varsigma_{\mathscr{O}} (\Delta_{i} )} } \right)^{2} \hfill \\ + \left( {\sqrt {\varsigma_{\mathscr{O}} (\Delta_{i} )} - \sqrt {\varsigma_{\mathscr{N}} (\Delta_{i} )} } \right)^{2} + \left( {\sqrt {\tau_{\mathscr{M}} (\Delta_{i} )} - \sqrt {\tau_{\mathscr{O}} (\Delta_{i} )} } \right)^{2} + \left( {\sqrt {\tau_{\mathscr{O}} (\Delta_{i} )} - \sqrt {\tau_{\mathscr{N}} (\Delta_{i} )} } \right)^{2} \hfill \\ + 2\left( {\sqrt {\sigma_{\mathscr{M}} (\Delta_{i} )} - \sqrt {\sigma_{\mathscr{O}} (\Delta_{i} )} } \right)\left( {\sqrt {\sigma_{\mathscr{O}} (\Delta_{i} )} - \sqrt {\sigma_{\mathscr{N}} (\Delta_{i} )} } \right) \hfill \\ + 2\left( {\sqrt {\varsigma_{\mathscr{M}} (\Delta_{i} )} - \sqrt {\varsigma_{\mathscr{O}} (\Delta_{i} )} } \right)\left( {\sqrt {\varsigma_{\mathscr{O}} (\Delta_{i} )} - \sqrt {\varsigma_{\mathscr{N}} (\Delta_{i} )} } \right) \hfill \\ + 2\left( {\sqrt {\tau_{\mathscr{M}} (\Delta_{i} )} - \sqrt {\tau_{\mathscr{O}} (\Delta_{i} )} } \right)\left( {\sqrt {\tau_{\mathscr{O}} (\Delta_{i} )} - \sqrt {\tau_{\mathscr{N}} (\Delta_{i} )} } \right) \hfill \\ \end{gathered} \right]} \\ \end{aligned} $$

$$ \begin{aligned} & = \mathscr{D}_{H}^{2} (\mathscr{M},\mathscr{O}) + \mathscr{D}_{H}^{2} (\mathscr{N},\mathscr{O}) + \frac{1}{{{3}\varvec{n}}}\sum\limits_{{\varvec{i} = 1}}^{\varvec{n}} {\left[ \begin{gathered} 2\left( {\sqrt {\sigma_{\mathscr{M}} (\Delta_{i} )} - \sqrt {\sigma_{\mathscr{O}} (\Delta_{i} )} } \right)\left( {\sqrt {\sigma_{\mathscr{O}} (\Delta_{i} )} - \sqrt {\sigma_{\mathscr{N}} (\Delta_{i} )} } \right) \hfill \\ + 2\left( {\sqrt {\varsigma_{\mathscr{M}} (\Delta_{i} )} - \sqrt {\varsigma_{\mathscr{O}} (\Delta_{i} )} } \right)\left( {\sqrt {\varsigma_{\mathscr{O}} (\Delta_{i} )} - \sqrt {\varsigma_{\mathscr{N}} (\Delta_{i} )} } \right) \hfill \\ + 2\left( {\sqrt {\tau_{\mathscr{M}} (\Delta_{i} )} - \sqrt {\tau_{\mathscr{O}} (\Delta_{i} )} } \right)\left( {\sqrt {\tau_{\mathscr{O}} (\Delta_{i} )} - \sqrt {\tau_{\mathscr{N}} (\Delta_{i} )} } \right) \hfill \\ \end{gathered} \right]} \\ & \le \mathscr{D}_{H}^{2} (\mathscr{M},\mathscr{O}) + \mathscr{D}_{H}^{2} (\mathscr{N},\mathscr{O}) + \frac{1}{{{3}\varvec{n}}}\left\{ \begin{gathered} 2\sqrt {\sum\limits_{{\varvec{i} = 1}}^{\varvec{n}} {\left( {\sqrt {\sigma_{\mathscr{M}} (\Delta_{i} )} - \sqrt {\sigma_{\mathscr{O}} (\Delta_{i} )} } \right)^{2} \times \sum\limits_{{\varvec{i} = 1}}^{\varvec{n}} {\left( {\sqrt {\sigma_{\mathscr{O}} (\Delta_{i} )} - \sqrt {\sigma_{\mathscr{N}} (\Delta_{i} )} } \right)^{2} } } } \hfill \\ + 2\sqrt {\sum\limits_{{\varvec{i} = 1}}^{\varvec{n}} {\left( {\sqrt {\varsigma_{\mathscr{M}} (\Delta_{i} )} - \sqrt {\varsigma_{\mathscr{O}} (\Delta_{i} )} } \right)^{2} \times \sum\limits_{{\varvec{i} = 1}}^{\varvec{n}} {\left( {\sqrt {\varsigma_{\mathscr{O}} (\Delta_{i} )} - \sqrt {\varsigma_{\mathscr{N}} (\Delta_{i} )} } \right)^{2} } } } \hfill \\ + \sqrt {\sum\limits_{{\varvec{i} = 1}}^{\varvec{n}} {\left( {\sqrt {\tau_{\mathscr{M}} (\Delta_{i} )} - \sqrt {\tau_{\mathscr{O}} (\Delta_{i} )} } \right)^{2} \times \sum\limits_{{\varvec{i} = 1}}^{\varvec{n}} {\left( {\sqrt {\tau_{\mathscr{O}} (\Delta_{i} )} - \sqrt {\tau_{\mathscr{N}} (\Delta_{i} )} } \right)^{2} } } } \hfill \\ \end{gathered} \right\} \\ & \le \mathscr{D}_{H}^{2} (\mathscr{M},\mathscr{O}) + \mathscr{D}_{H}^{2} (\mathscr{N},\mathscr{O}) \\ & \quad + 2 \times \frac{1}{{{3}\varvec{n}}}\sqrt {\sum\limits_{{\varvec{i} = 1}}^{\varvec{n}} {\left( {\sqrt {\sigma_{\mathscr{M}} (\Delta_{i} )} - \sqrt {\sigma_{\mathscr{O}} (\Delta_{i} )} } \right)^{2} + \sum\limits_{{\varvec{i} = 1}}^{\varvec{n}} {\left( {\sqrt {\varsigma_{\mathscr{M}} (\Delta_{i} )} - \sqrt {\varsigma_{\mathscr{O}} (\Delta_{i} )} } \right)^{2} } + \sum\limits_{{\varvec{i} = 1}}^{\varvec{n}} {\left( {\sqrt {\tau_{\mathscr{M}} (\Delta_{i} )} - \sqrt {\tau_{\mathscr{O}} (\Delta_{i} )} } \right)^{2} } } } \\ & \quad \times \sqrt {\sum\limits_{{\varvec{i} = 1}}^{\varvec{n}} {\left( {\sqrt {\sigma_{\mathscr{O}} (\Delta_{i} )} - \sqrt {\sigma_{\mathscr{N}} (\Delta_{i} )} } \right)^{2} + \sum\limits_{{\varvec{i} = 1}}^{\varvec{n}} {\left( {\sqrt {\varsigma_{\mathscr{O}} (\Delta_{i} )} - \sqrt {\varsigma_{\mathscr{N}} (\Delta_{i} )} } \right)^{2} } + \sum\limits_{{\varvec{i} = 1}}^{\varvec{n}} {\left( {\sqrt {\tau_{\mathscr{O}} (\Delta_{i} )} - \sqrt {\tau_{\mathscr{N}} (\Delta_{i} )} } \right)^{2} } } } \\ & = \mathscr{D}_{H}^{2} (\mathscr{M},\mathscr{O}) + \mathscr{D}_{H}^{2} (\mathscr{N},\mathscr{O}) + 2\mathscr{D}_{H} (\mathscr{M},\mathscr{O})\mathscr{D}_{H} (\mathscr{N},\mathscr{O}) \\ & = \left[ {\mathscr{D}_{H} (\mathscr{M},\mathscr{O}) + \mathscr{D}_{H} (\mathscr{N},\mathscr{O})} \right]^{2} . \\ \end{aligned} $$

Therefore, $\mathscr{D}(\mathscr{M},\mathscr{N}) \le \mathscr{D}(\mathscr{M},\mathscr{O}) + \mathscr{D}(\mathscr{O},\mathscr{N})$. Then the theorem is proved.

#### Theorem 2

Let $\mathscr{T} = \{ \Delta_{1} ,\Delta_{2} ,...,\Delta_{\varvec{n}} \}$ be a given domain, and $w_{i}$ is the degree of importance of $\Delta_{i}$. There are two SF sets $\mathscr{M} = \{ \langle \Delta_{i} ,\sigma_{\mathscr{M}} (\Delta_{i} ),\varsigma_{\mathscr{M}} (\Delta_{i} ),\tau_{\mathscr{M}} (\Delta_{i} )\rangle \left| {\Delta_{i} \in \mathscr{T}} \right.\}$ and $\mathscr{N} = \{ \langle \Delta_{i} ,\sigma_{\mathscr{N}} (\Delta_{i} ),\varsigma_{\mathscr{N}} (\Delta_{i} ),\tau_{\mathscr{N}} (\Delta_{i} )\rangle \left| {\Delta_{i} \in \mathscr{T}} \right.\}$ defined in $\mathscr{T}$. Then we can obtain a new weighted distance between $\mathscr{M}$ and $\mathscr{N}$ as follows:

16 $$ \mathscr{D}_{HW} (\mathscr{M},\mathscr{N}) = \sqrt {\frac{1}{{3}}\sum\limits_{{\varvec{i} = 1}}^{\varvec{n}} {w_{i} \left( {\sqrt {\sigma_{\mathscr{M}} (\Delta_{\varvec{i}} )} - \sqrt {\sigma_{\mathscr{N}} (\Delta_{\varvec{i}} )} } \right)^{2} + \left( {\sqrt {\varsigma_{\mathscr{M}} (\Delta_{\varvec{i}} )} - \sqrt {\varsigma_{\mathscr{N}} (\Delta_{\varvec{i}} )} } \right)^{2} + \left( {\sqrt {\tau_{\mathscr{M}} (\Delta_{\varvec{i}} )} - \sqrt {\tau_{\mathscr{N}} (\Delta_{\varvec{i}} )} } \right)^{2} } } $$

The proof of Theorem 2 is similar to the proof of Theorem 1 and is omitted here.

### Comparative analysis

To facilitate analysis and comparison, the existing formulas for SF distance formulas are listed below.

Let $\mathscr{M} = \{ \langle \Delta ,\sigma _{\mathscr{M}} (\Delta ),\varsigma _{\mathscr{M}} (\Delta ),\tau _{\mathscr{M}} (\Delta )\rangle \left| {\Delta  \in \mathscr{T}} \right.\}$ and $\mathscr{N} = \{ \langle \Delta ,\sigma _{\mathscr{N}} (\Delta ),\varsigma _{\mathscr{N}} (\Delta ),\tau _{\mathscr{N}} (\Delta )\rangle \left| {\Delta  \in \mathscr{T}} \right.\}$ be two SF sets defined in the domain $\mathscr{T} = \{ \Delta_{1} ,\Delta_{2} ,...,\Delta_{\varvec{n}} \}$. Ashraf et al.^49, 50^ proposed the following distance formulas:

17 $$ \mathscr{D}_{aa1} (\mathscr{M},\mathscr{N}) = \frac{1}{\varvec{n}}\sum\limits_{{\varvec{i} = 1}}^{\varvec{n}} {\left[ {|\sigma_{\mathscr{M}} (\Delta_{\varvec{i}} ) - \sigma_{\mathscr{N}} (\Delta_{\varvec{i}} )| + |\varsigma_{\mathscr{M}} (\Delta_{\varvec{i}} ) - \varsigma_{\mathscr{N}} (\Delta_{\varvec{i}} )| + |\tau_{\mathscr{M}} (\Delta_{\varvec{i}} ) - \tau_{\mathscr{N}} (\Delta_{\varvec{i}} )|} \right]} , $$

18 $$ \mathscr{D}_{aa2} (\mathscr{M},\mathscr{N}) = \frac{1}{\varvec{n}}\sum\limits_{{\varvec{i} = 1}}^{\varvec{n}} {\left[ {|\sigma_{\mathscr{M}} (\Delta_{\varvec{i}} ) - \sigma_{\mathscr{N}} (\Delta_{\varvec{i}} )|^{2} + |\varsigma_{\mathscr{M}} (\Delta_{\varvec{i}} ) - \varsigma_{\mathscr{N}} (\Delta_{\varvec{i}} )|^{2} + |\tau_{\mathscr{M}} (\Delta_{\varvec{i}} ) - \tau_{\mathscr{N}} (\Delta_{\varvec{i}} )|^{2} } \right]} , $$

19 $$ \mathscr{D}_{al1} (\mathscr{M},\mathscr{N}) = \frac{1}{\varvec{n}}\sum\limits_{{\varvec{i} = 1}}^{\varvec{n}} {\max \{ |\sigma_{\mathscr{M}} (\Delta_{\varvec{i}} ) - \sigma_{\mathscr{N}} (\Delta_{\varvec{i}} )|,|\varsigma_{\mathscr{M}} (\Delta_{\varvec{i}} ) - \varsigma_{\mathscr{N}} (\Delta_{\varvec{i}} )|,|\tau_{\mathscr{M}} (\Delta_{\varvec{i}} ) - \tau_{\mathscr{N}} (\Delta_{\varvec{i}} )|\} } , $$

20 $$ \mathscr{D}_{al2} (\mathscr{M},\mathscr{N}) = \frac{1}{\varvec{n}}\sum\limits_{{\varvec{i} = 1}}^{\varvec{n}} {\min \{ |\sigma_{\mathscr{M}} (\Delta_{\varvec{i}} ) - \sigma_{\mathscr{N}} (\Delta_{\varvec{i}} )|,|\varsigma_{\mathscr{M}} (\Delta_{\varvec{i}} ) - \varsigma_{\mathscr{N}} (\Delta_{\varvec{i}} )|,|\tau_{\mathscr{M}} (\Delta_{\varvec{i}} ) - \tau_{\mathscr{N}} (\Delta_{\varvec{i}} )|\} } , $$

21 $$ \mathscr{D}_{al3} (\mathscr{M},\mathscr{N}) = \frac{1}{\varvec{n}}\sum\limits_{{\varvec{i} = 1}}^{\varvec{n}} {\left[ {|\sigma_{\mathscr{M}} (\Delta_{\varvec{i}} ) - \sigma_{\mathscr{N}} (\Delta_{\varvec{i}} )|^{\rho } + |\varsigma_{\mathscr{M}} (\Delta_{\varvec{i}} ) - \varsigma_{\mathscr{N}} (\Delta_{\varvec{i}} )|^{\rho } + |\tau_{\mathscr{M}} (\Delta_{\varvec{i}} ) - \tau_{\mathscr{N}} (\Delta_{\varvec{i}} )|^{\rho } } \right]} , $$

Here $$\rho \ge 1$$.

The distance formula of Mahood et al.^51^:

22 $$ \mathscr{D}_{mu} (\mathscr{M},\mathscr{N}) = \frac{1}{\varvec{n}}\sum\limits_{{\varvec{i} = 1}}^{\varvec{n}} {\left[ {|\sigma_{\mathscr{M}}^{2} (\Delta_{\varvec{i}} ) - \sigma_{\mathscr{N}}^{2} (\Delta_{\varvec{i}} )| + |\varsigma_{\mathscr{M}}^{2} (\Delta_{\varvec{i}} ) - \varsigma_{\mathscr{N}}^{2} (\Delta_{\varvec{i}} )| + |\tau_{\mathscr{M}}^{2} (\Delta_{\varvec{i}} ) - \tau_{\mathscr{N}}^{2} (\Delta_{\varvec{i}} )|} \right]} , $$

The distance formula of Khan et al.^52^:

23 $$ \mathscr{D}_{kk} (\mathscr{M},\mathscr{N}) = \frac{{\sum\limits_{{\varvec{i} = 1}}^{\varvec{n}} {\left[ {\sigma_{\mathscr{M}}^{2} (\Delta_{\varvec{i}} )\sigma_{\mathscr{N}}^{2} (\Delta_{\varvec{i}} ) + \varsigma_{\mathscr{M}}^{2} (\Delta_{\varvec{i}} )\varsigma_{\mathscr{N}}^{2} (\Delta_{\varvec{i}} ) + \tau_{\mathscr{M}}^{2} (\Delta_{\varvec{i}} )\tau_{\mathscr{N}}^{2} (\Delta_{\varvec{i}} )} \right]} }}{{\sum\limits_{{\varvec{i} = 1}}^{\varvec{n}} {\left[ {\sigma_{\mathscr{M}}^{2} (\Delta_{\varvec{i}} ) \vee \sigma_{\mathscr{N}}^{2} (\Delta_{\varvec{i}} ) + \varsigma_{\mathscr{M}}^{2} (\Delta_{\varvec{i}} ) \vee \varsigma_{\mathscr{N}}^{2} (\Delta_{\varvec{i}} ) + \tau_{\mathscr{M}}^{2} (\Delta_{\varvec{i}} ) \vee \tau_{\mathscr{N}}^{2} (\Delta_{\varvec{i}} )} \right]} }}, $$

The distance formula of Ali and Garg^40^:

24 $$\mathscr{D}_{{JH}} (\mathscr{M},\mathscr{N}) = \frac{1}{{4\sqrt {\varvec{n}} }}\sum\limits_{{\varvec{i} = 1}}^{\varvec{n}} {\left[ \begin{gathered}   ||u(\sigma _{\mathscr{M}} (\Delta _{\varvec{i}} )) - u(\sigma _{\mathscr{N}} (\Delta _{\varvec{i}} ))|| + ||u(\varsigma _{\mathscr{M}} (\Delta _{\varvec{i}} )) - u(\varsigma _{\mathscr{N}} (\Delta _{\varvec{i}} ))|| \hfill \\    + ||u(\tau _{\mathscr{M}} (\Delta _{\varvec{i}} )) - u(\tau _{\mathscr{N}} (\Delta _{\varvec{i}} ))|| + ||u(\pi _{\mathscr{M}} (\Delta _{\varvec{i}} )) - u(\pi _{\mathscr{N}} (\Delta _{\varvec{i}} ))|| \hfill \\  \end{gathered}  \right]} ,  $$

where

25 $$ \begin{aligned} u(\sigma_{\mathscr{M}} (\Delta )) & = [F(\sigma_{\mathscr{M}} (\Delta_{\varvec{i}} ),\sigma_{\mathscr{M}} (\Delta_{\varvec{j}} ))]_{{\varvec{n} \times \varvec{n}}} \\ & = \left[ {\begin{array}{*{20}c} {F(\sigma_{\mathscr{M}} (\Delta_{1} ),\sigma_{\mathscr{M}} (\Delta_{1} ))} & \cdots & {F(\sigma_{\mathscr{M}} (\Delta_{1} ),\sigma_{\mathscr{M}} (\Delta_{\varvec{n}} ))} \\ \vdots & \ddots & \vdots \\ {F(\sigma_{\mathscr{M}} (\Delta_{\varvec{n}} ),\sigma_{\mathscr{M}} (\Delta_{1} ))} & \ldots & {F(\sigma_{\mathscr{M}} (\Delta_{\varvec{n}} ),\sigma_{\mathscr{M}} (\Delta_{\varvec{n}} ))} \\ \end{array} } \right], \\ \end{aligned} $$

26 $$ \begin{aligned} u(\varsigma_{\mathscr{M}} (\Delta )) & = [F(\varsigma_{\mathscr{M}} (\Delta_{\varvec{i}} ),\varsigma_{\mathscr{M}} (\Delta_{\varvec{j}} ))]_{{\varvec{n} \times \varvec{n}}} \\ & = \left[ {\begin{array}{*{20}c} {F(\varsigma_{\mathscr{M}} (\Delta_{1} ),\varsigma_{\mathscr{M}} (\Delta_{1} ))} & \cdots & {F(\varsigma_{\mathscr{M}} (\Delta_{1} ),\varsigma_{\mathscr{M}} (\Delta_{\varvec{n}} ))} \\ \vdots & \ddots & \vdots \\ {F(\varsigma_{\mathscr{M}} (\Delta_{\varvec{n}} ),\varsigma_{\mathscr{M}} (\Delta_{1} ))} & \ldots & {F(\varsigma_{\mathscr{M}} (\Delta_{\varvec{n}} ),\varsigma_{\mathscr{M}} (\Delta_{\varvec{n}} ))} \\ \end{array} } \right], \\ \end{aligned} $$

27 $$ \begin{aligned} u(\tau_{\mathscr{M}} (\Delta )) & = [F(\tau_{\mathscr{M}} (\Delta_{\varvec{i}} ),\tau_{\mathscr{M}} (\Delta_{\varvec{j}} ))]_{{\varvec{n} \times \varvec{n}}} \\ & = \left[ {\begin{array}{*{20}c} {F(\tau_{\mathscr{M}} (\Delta_{1} ),\tau_{\mathscr{M}} (\Delta_{1} ))} & \cdots & {F(\tau_{\mathscr{M}} (\Delta_{1} ),\tau_{\mathscr{M}} (\Delta_{\varvec{n}} ))} \\ \vdots & \ddots & \vdots \\ {F(\tau_{\mathscr{M}} (\Delta_{\varvec{n}} ),\tau_{\mathscr{M}} (\Delta_{1} ))} & \ldots & {F(\tau_{\mathscr{M}} (\Delta_{\varvec{n}} ),\tau_{\mathscr{M}} (\Delta_{\varvec{n}} ))} \\ \end{array} } \right], \\ \end{aligned} $$

28 $$ \begin{aligned} u(\pi_{\mathscr{M}} (\Delta )) & = [F(\pi_{\mathscr{M}} (\Delta_{\varvec{i}} ),\pi_{\mathscr{M}} (\Delta_{\varvec{j}} ))]_{{\varvec{n} \times \varvec{n}}} \\ & = \left[ {\begin{array}{*{20}c} {F(\pi_{\mathscr{M}} (\Delta_{1} ),\pi_{\mathscr{M}} (\Delta_{1} ))} & \cdots & {F(\pi_{\mathscr{M}} (\Delta_{1} ),\pi_{\mathscr{M}} (\Delta_{\varvec{n}} ))} \\ \vdots & \ddots & \vdots \\ {F(\pi_{\mathscr{M}} (\Delta_{\varvec{n}} ),\pi_{\mathscr{M}} (\Delta_{1} ))} & \ldots & {F(\pi_{\mathscr{M}} (\Delta_{\varvec{n}} ),\pi_{\mathscr{M}} (\Delta_{\varvec{n}} ))} \\ \end{array} } \right]. \\ \end{aligned} $$

Here, $F:[0,1] \times [0,1] \to [0,1]$ is a strictly monotonic increasing or decreasing function, $\left\| u \right\| = \sqrt {\varsigma_{\max } }$, and $\varsigma_{\max }$ is the largest non-negative eigenvalue of a positive definite Hermitian matrix $u^{T} u$.

#### Example 1

To further examine the validity of the SF distance function proposed in this article, we will list five pairs of special SF sets (numbers) and calculate their distances. The cases of SFNs is shown in Table  2 and the results are shown in Table 3, where bold font is used to indicate counterintuitive situations. Here, $\rho = 2$ in $\mathscr{D}_{al3} (\mathscr{M},\mathscr{N})$ and $F\left( {x_{1} ,x_{2} } \right)$ in $\mathscr{D}_{JH} (\mathscr{M},\mathscr{N})$.

**Table 2:** The cases of SFNs.

Case	$\mathscr{M}$	$\mathscr{N}$
Case1	< 0.5,0.3,0.6 >	< 0.4,0.2,0.6 >
Case2	< 0.4,0.3,0.5 >	< 0.3,0.2,0.5 >
Case3	< 0.4,0.3,0.5 >	< 0.5,0.4,0.5 >
Case4	< 0.4,0.2,0.3 >	< 0.2,0.4,0.3 >
Case5	$< {0}{\text{.3,}}\;\sqrt {{0}{\text{.21}}} {,}\;{0}{\text{.6}} >$	$< \sqrt {{0}{\text{.21}}} {,0}{\text{.3,0}}{.6} >$

**Table 3:** Distance between SF numbers A and B in five cases.

Case	$\mathscr{D}_{aa1}$	$\mathscr{D}_{aa2}$	$\mathscr{D}_{al1}$	$\mathscr{D}_{al2}$	$\mathscr{D}_{al3}$	$\mathscr{D}_{mu}$	$\mathscr{D}_{kk}$	$\mathscr{D}_{JH}$	$\mathscr{D}_{H}$
Case 1	**0.2000**	**0.1414**	**0.1000**	**0.0000**	**0.1414**	0.1400	0.1349	0.0700	0.0723
Case 2	**0.2000**	**0.1414**	**0.1000**	**0.0000**	**0.1414**	0.1200	0.1632	0.0698	0.0759
Case 3	**0.2000**	**0.1414**	**0.1000**	**0.0000**	**0.1414**	0.1600	0.2238	0.0800	0.0652
Case 4	0.4000	0.2828	0.2000	**0.0000**	0.2828	**0.2400**	0.6476	0.0600	0.1512
Case 5	0.3165	0.4899	0.1583	**0.0000**	0.4899	**0.2400**	0.2314	0.0655	0.1055

As shown in Table 3, it can be seen that $\mathscr{D}_{kk}$, $\mathscr{D}_{JH}$ and the SF Hellinger distance measure $\mathscr{D}_{H}$ are more reasonable than other distance measures. However, the distance measure $\mathscr{D}_{kk}$ cannot handle the case where the denominator is zero, and the calculation of the distance measure $\mathscr{D}_{JH}$ is too complex. In contrast, the distance measure proposed in this article is simple and fast to calculate.

## New spherical fuzzy MADM method based on Helinger distance and regret theory

### A new spherical fuzzy MADM model

For a decision making problem, if the attribute evaluation values or attribute weight information contains SF numbers, then the problem can be regarded as a SF MADM problem. For ease of description, the following symbols represent sets or quantities in the assessment process:

$$A_{i}$$
The *i*-th alternative;
$$o_{j}$$
The *j-*th attribute;
$$w_{j}$$
The importance level of the *i*-th attribute $o_{j}$. Obviously $0 \le \omega_{j} \le 1\left( {j = 1,2, \ldots m} \right)$ and $\sum\nolimits_{j = 1}^{m} {w_{j} = 1}$;
$$x_{ij} = < \mu_{ij} ,\eta_{ij} ,\upsilon_{ij} >$$
The attribute value of solution $A_{i}$ under attribute $o_{j}$ is a SF number.

Therefore, the decision information matrix for this assessment problem of logistics providers is $\tilde{\user2{D}} = ( < \mu_{ij} ,\eta_{ij} ,\upsilon_{ij} > )_{m \times n}$.

In practical assessment process, the DM often directly provides the weights of evaluation attributes. However, there are also situations where the DM may not be able to accurately provide the weights of evaluation attributes due to their knowledge background and familiarity with the problem. Sometimes, they may have no information about attribute weights, while in other cases, they can only provide partial information about attribute weights. Let ${\varvec{H}}$ be the set of mathematical expressions representing known partial attribute weight information.

In MADM model, determining the weights of decision attributes is a very important research topic. There are already many methods for determining attribute weights based on information measures, such as entropy weight method, maximum deviation method, and optimization model solving methods based on information measures^53–58^. How to measure its fuzziness and how to measure the distance and discrimination between two SF sets well need to construct reasonable distance measure, similarity and other information measures to solve these problems. At the same time, these information measures mentioned above also help to determine attribute weights. Some scholars have constructed SF information measures and developed attribute weight determination methods and MADM methods. Ayodou and Gül^59^ constructed a new type of SF entropy measure and used the entropy weight method to determine attribute weights. They developed the SF weighted sum product evaluation method based on the new entropy measure. By comparing and analyzing the decision results obtained with other methods, it was found that the new decision method is more robust. Ashraf et al.^49^ defined the Euclidean distance and Hamming distance of SF set, and constructed the weighted operator of fuzzy distance based on these two distances. In this section, we will develop a new model (Model (37)) to help solve the attribute weights.

### New MADM method based on regret theory

The regret theory is a psychological theory that explores how people consider the potential consequences and feelings of regret when making decisions. The theory suggests that when making decisions, people not only consider current benefits and risks but also anticipate future outcomes and predict whether they will feel regret as a result.

The perceived utility value (PUV) of DM is defined as follows:

29 $$ \mathscr{G}(\alpha ,\beta ) = V(\alpha ) + \mathscr{R}(V(\alpha ) - V(\beta )) $$

where $\alpha$ and $\beta$ are the results that can be obtained by selecting two alternatives $A$ and $B$, respectively. Among them, $V(\alpha )$ and $V(\beta )$ respectively represent the utility value $A$ and $B$ after the DM selects the scheme and $\mathscr{R}(V(\alpha ) - V(\beta ))$ is called regret-rejoice value, and if it is positive, we called it the rejoice value. Otherwise, it is called the regret value. Loomes and Sugden^25^ suggested that $\mathscr{R}( \cdot )$ can be chosen in the following function form:

30 $$  \mathscr{R}(\tau ) = 1 - e^{ - \xi \tau } $$

where, $\xi > 0$ is the regret avoidance coefficient, and $\tau$ is the difference between $V(\alpha )$ and $V(\beta )$.

Based on regret theory, when the positive ideal solution (PIS) is chosen as the reference point, the decision evaluation value of other options cannot be higher than the decision evaluation value of the PIS, and the DM will feel regretful; when the negative ideal solution (NIS) is chosen as the reference point, the decision evaluation value of other options cannot be lower than the decision evaluation value of the NIS, and the DM will feel happy.

Let $x_{ij}$ be the attribute value of alternative $A_{i}$ under attribute $o_{j}$, then the regret value of $x_{ij}$ is relative to the corresponding attribute value $x_{j}^{ + }$ of the PIS and $x_{j}^{ - }$ of NIS are defined as follows^28^:

31 $$  \mathscr{R}_{ij}^{1} = 1 - e^{{\xi |x_{ij} - x_{j}^{ + } {|}}} $$

32 $$  \mathscr{R}_{ij}^{2} = 1 - e^{{ - \xi |x_{ij} - x_{j}^{ + } {|}}} $$

Then, the comprehensive regret-rejoice value for $x_{ij}$ of $A_{i}$ under $o_{j}$ is

33 $$  \mathscr{R}_{ij} =  \mathscr{R}_{ij}^{1} +  \mathscr{R}_{ij}^{2} = 2 - e^{{\xi |x_{ij} - x_{j}^{ + } {|}}} - e^{{ - \xi |x_{ij} - x_{j}^{ + } {|}}} $$

According to Bell^24^, the power function $V_{ij} (x) = x^{\theta } , - \infty < x < \infty ,0 < \theta < 1$ can be used as a utility function, where $\theta$ is the risk aversion coefficient of DMs.

This section will construct a new regret theory and Hellinger distance-based SF MADM model. Assume that the attribute evaluation value of $A_{i}$ under $o_{j}$ be a SF number $x_{ij} = < \sigma_{ij} ,\varsigma_{ij} ,\tau_{ij} >$.

In this study, a function $V(x_{ij} ) = (S(x_{ij} ))^{\theta }$ is used as a utility function of $x_{ij} = < \sigma_{ij} ,\varsigma_{ij} ,\tau_{ij} >$. Then, the DM’s perception utility value (PUV) of SF number $x_{ij}$ of the scheme $A_{i}$ can be defined as:

34 $$ F_{ij} = V_{ij} +  \mathscr{R}_{ij} = 2 + (S(x_{ij} ))^{\theta } - e^{{\xi d(x_{ij} ,x_{j}^{ + } )}} - e^{{ - \xi d(x_{ij} ,x_{j}^{ + } )}} $$

Here, $d(\varvec{x},\varvec{y})$ represents the Hellinger distance between two SF numbers $\varvec{x}$ and $\varvec{y}$. $S(x_{ij} )$ is the score function, which is defined as follows (Ali^60^):

35 $$ S_{ij} = \sigma_{ij}^{2} - \varsigma_{ij}^{2} - \ln (1 + \pi_{ij}^{2} ), $$

where $$\pi_{ij} = 1 - \sigma_{ij}^{2} - \varsigma_{ij}^{2} - \tau_{ij}^{2}$$.

Next, we will discuss the method for determining attribute weights when partial weight information is known. Let the set of known weight information be denoted as ${\varvec{H}}$. For each $A_{i}$, its overall PUV is denoted as

36 $$ \Gamma (A_{i} ) = \sum\limits_{j = 1}^{n} {w_{j} F_{ij} } = \sum\limits_{j = 1}^{n} {w_{j} \left( {2 + (S(x_{ij} ))^{\theta } - e^{{\xi d(x_{ij} ,x_{j}^{ + } )}} - e^{{ - \xi d(x_{ij} ,x_{j}^{ + } )}} } \right)} $$

The determination of weights should aim to maximize the overall PUV of each scheme $A_{i}$. That is, the objective function is $\max \;\Gamma = (\Gamma (A_{1} ),\Gamma (A_{2} ), \cdots ,\Gamma (A_{m} ))$.

Based on the principle that “the larger the overall PUV, the better the scheme”, and assuming fair competition among all alternatives, the following optimization model (37) is established with the goal of maximizing the DM’s overall PUV of the alternative set.

37 $$ \begin{gathered} \max \sum\limits_{i = 1}^{m} {\Gamma (A_{i} )} = \sum\limits_{i = 1}^{m} {\sum\limits_{j = 1}^{n} {w_{j} F_{ij} } } = \sum\limits_{i = 1}^{m} {\sum\limits_{j = 1}^{n} {w_{j} \left( {2 + (S(x_{ij} ))^{\theta } - e^{{\xi d(x_{ij} ,x_{j}^{ + } )}} - e^{{ - \xi d(x_{ij} ,x_{j}^{ + } )}} } \right)} } \hfill \\ s.t.\;\left\{ \begin{gathered} {\varvec{w}} \in {\varvec{H}} \hfill \\ \sum\limits_{j = 1}^{n} {w_{j} = 1} \hfill \\ w_{1} ,w_{2} , \ldots ,w_{n} \ge 0 \hfill \\ \end{gathered} \right. \hfill \\ \end{gathered} $$

By solving the above model we can solve the optimal weight vector ${\varvec{w}}^{ * } \user2{ = }(w_{1}^{ * } ,w_{2}^{ * } , \cdots ,w_{n}^{ * } )$.

The optimal overall PUV for $A_{i}$ is

38 $$ PUV(A_{i} ) = \sum\limits_{j = 1}^{n} {w_{j}^{ * } \,\left( {2 + (S(x_{ij} ))^{\alpha } - e^{{\xi d(x_{ij} ,x_{j}^{ + } )}} - e^{{ - \xi d(x_{ij} ,x_{j}^{ + } )}} } \right)} $$

**Figure 2:**
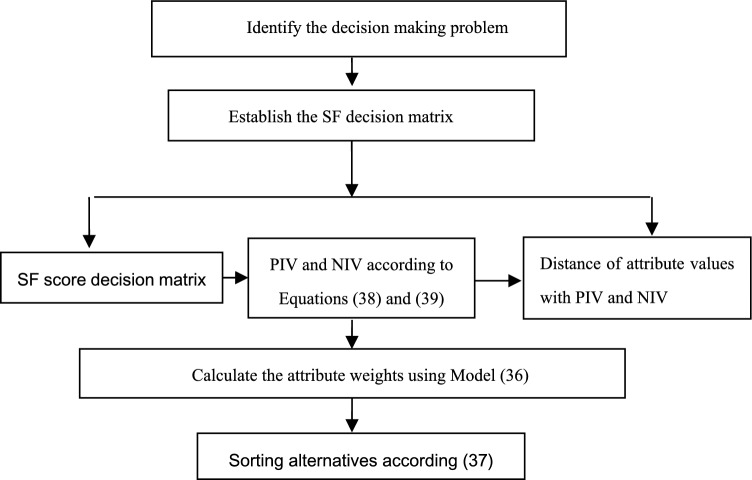
Steps of our proposed SF algorithm.

Figure 2 illustrates steps of our proposed algorithm for solving MADM problems.

In the follow, we introduce the steps for SF MADM method based on the new hellinger distance and regret theory.

*Step 1.* Identify the problem and establish the SF decision matrix $\tilde{\user2{D}} = ( < \sigma_{ij} ,\varsigma_{ij} ,\tau_{ij} > )_{m \times n}$.

*Step 2.* According to Eq. (35), calculate the scores of $x_{ij} = < \sigma_{ij} ,\varsigma_{ij} ,\tau_{ij} >$ , then we get the SF score matrix $\user2{S = }(S_{ij} )_{m \times n}$.

*Step 3.* Define the positive ideal value (PIV) and negative ideal value (NIV) of attribute $o_{j}$.

PIV $x_{j}^{ + }$ is defined as:

39 $$ \begin{aligned} x_{j}^{ + } & = < \sigma_{j}^{ + } ,\varsigma_{j}^{ + } ,\tau_{j}^{ + } > \\ & = \left\{ \begin{gathered} \mathop {\max }\limits_{1 \le i \le n} \{ x_{ij} \} ,\;o_{j} \in BB \hfill \\ \mathop {\min }\limits_{1 \le i \le n} \{ x_{ij} \} ,\;o_{j} \in CC \hfill \\ \end{gathered} \right. \\ & = \left\{ \begin{gathered} < \mathop {\max }\limits_{1 \le i \le n} \{ \sigma_{ij} \} ,\mathop {\max }\limits_{1 \le i \le n} \{ \varsigma_{ij} \} ,\mathop {\min }\limits_{1 \le i \le n} \{ \tau_{ij} \} > ,\;o_{j} \in BB \hfill \\ < \mathop {\min }\limits_{1 \le i \le n} \{ \sigma_{ij} \} ,\mathop {\min }\limits_{1 \le i \le n} \{ \varsigma_{ij} \} ,\mathop {\max }\limits_{1 \le i \le n} \{ \tau_{ij} \} > ,\;o_{j} \in CC, \hfill \\ \end{gathered} \right. \\ \end{aligned} $$

NIV $x_{j}^{ - }$ is defined as:

40 $$ \begin{aligned} x_{j}^{ - } & = < \sigma_{j}^{ - } ,\varsigma_{j}^{ - } ,\tau_{j}^{ - } > \\ & = \left\{ \begin{gathered} \mathop {\min }\limits_{1 \le i \le n} \{ x_{ij} \} ,\;o_{j} \in BB \hfill \\ \mathop {\max }\limits_{1 \le i \le n} \{ x_{ij} \} ,\;o_{j} \in CC \hfill \\ \end{gathered} \right. \\ & = \left\{ \begin{gathered} < \mathop {\min }\limits_{1 \le i \le n} \{ \sigma_{ij} \} ,\mathop {\min }\limits_{1 \le i \le n} \{ \varsigma_{ij} \} ,\mathop {\max }\limits_{1 \le i \le n} \{ \tau_{ij} \} > ,\;o_{j} \in BB \hfill \\ < \mathop {\max }\limits_{1 \le i \le n} \{ \sigma_{ij} \} ,\mathop {\max }\limits_{1 \le i \le n} \{ \varsigma_{ij} \} ,\mathop {\min }\limits_{1 \le i \le n} \{ \tau_{ij} \} > ,\;o_{j} \in CC \hfill \\ \end{gathered} \right., \\ \end{aligned} $$

where $BB$ represents the set of benefit-type indicators and $CC$ represents the set of cost-type indicators.

*Step 4.* Calculate the attribute values of each alternative (i.e. $x_{ij} = < \sigma_{ij} ,\varsigma_{ij} ,\tau_{ij} >$) and the corresponding distances to the PIV and NIV, respectively. According to Eq. (4), we have:

41 $$ \mathscr{D}_{H} (x_{ij} ,x_{j}^{ + } ) = \sqrt {\frac{{\left( {\sqrt {\mu_{ij} } - \sqrt {\mu_{j}^{ + } } } \right)^{2} + \left( {\sqrt {\eta_{ij} } - \sqrt {\eta_{j}^{ + } } } \right)^{2} + \left( {\sqrt {\upsilon_{ij} } - \sqrt {\upsilon_{j}^{ + } } } \right)^{2} }}{3}} , $$

42 $$ \mathscr{D}_{H} \left( {x_{ij} ,x_{j}^{ - } } \right) = \sqrt {\frac{{\left( {\sqrt {\mu_{ij} } - \sqrt {\mu_{j}^{ - } } } \right)^{2} + \left( {\sqrt {\eta_{ij} } - \sqrt {\eta_{j}^{ - } } } \right)^{2} + \left( {\sqrt {\upsilon_{ij} } - \sqrt {\upsilon_{j}^{ - } } } \right)^{2} }}{3}} . $$

*Step 5.* Substitute Eqs. (38), (41), and (42) into Eq. (36) to calculate the overall PUV $\Gamma (A_{i} )$ for each alternative , respectively.

*Step 6.* With the goal of maximizing the overall PUVs of the DM for the set of alternatives, construct the optimization model (37).

*Step 7.* Substitute the optimal attribute weights obtained from Step 6 into Eq. (38) to obtain the overall PUV for each candidate alternative. The superiority or inferiority of each alternative is determined by the value of $PUV(A_{i} )$. The larger the value of $PUV(A_{i} )$, the better the corresponding candidate alternative $A_{i}$.

## Application of assessment of logistics provider

### Index system of assessment of 4PL providers

The evaluation index system of fourth-party logistics (4PL) providers is an important task for enterprises when choosing 4PL service providers. After referring to the literature on the evaluation index system of logistics suppliers, it is found that the evaluation index system is not unified. Based on the need of case analysis and the summary of existing literature, the index system mainly includes aspects such as service quality, cost-effectiveness, technical ability, management system, and innovation ability^61–63^.

(i) *Service quality* Service quality is one of the most important factors that enterprises pay attention to when choosing 4PL providers. Service quality includes indicators such as delivery time, delivery accuracy, damage rate, and customer satisfaction.
(ii) *Cost-effectiveness* Cost-effectiveness is another important factor that enterprises need to consider when choosing 4PL providers. Cost-effectiveness includes indicators such as transportation costs, inventory costs, and order processing costs.
(iii) *Technical ability* Technical ability is another important factor that enterprises need to consider when choosing 4PL providers. Technical ability includes indicators such as information technology level, logistics management ability, and transportation equipment level.
(iv) *Management system* The management system is another important factor that enterprises need to consider when choosing 4PL providers. The management system includes indicators such as quality management system, environmental management system, and safety management system.
(v) *Innovation ability* Innovation ability is another important factor that enterprises need to consider when choosing 4PL providers. Innovation ability includes indicators such as new product development ability and innovation management ability.

These indicators can be adjusted and supplemented according to actual conditions. By constructing the evaluation index system of 4PL providers, enterprises can more comprehensively understand the performance of 4PL providers and choose the most suitable provider to improve logistics efficiency and reduce costs.

### Numerical example of assessment of logistics providers

#### Example 2

A company wants to select the most suitable 4PL service provider from four candidates, and after expert discussions, six evaluation attributes are determined: cost-effectiveness ($o_{1}$), Service quality ($o_{2}$), technical ability ($o_{3}$), management system ($o_{4}$), innovation ability ($o_{5}$). Based on the five evaluation attributes, assuming that after discussions with experts and the leadership team, a SF decision matrix is established for decision-making judgments on the four candidate solutions, as shown in Table 4.

**Table 4:** Evaluation attribute values of each 4PL service providers.

	$o_{1}$	$o_{2}$	$o_{3}$	$o_{4}$	$o_{5}$
$A_{1}$	< 0.2, 0.8, 0.1 >	< 0.6, 0.4, 0.4 >	< 0.5, 0.5, 0.4 >	< 0.6, 0.1, 0.1 >	< 0.6, 0.4, 0.4 >
$A_{2}$	< 0.4, 0.6, 0.3 >	< 0.5, 0.4, 0.4 >	< 0.5, 0.4, 0.4 >	< 0.6, 0.2, 0.1 >	< 0.9, 0.0, 0.1 >
$A_{3}$	< 0.6, 0.3, 0.4 >	< 0.9, 0.1, 0.1 >	< 0.6, 0.4, 0.4 >	< 0.9, 0.1, 0.0 >	< 0.6, 0.4, 0.3 >
$A_{4}$	< 0.3, 0.7, 0.3 >	< 0.3, 0.6, 0.3 >	< 0.4, 0.5, 0.4 >	< 0.6, 0.1, 0.0 >	< 0.6, 0.3, 0.4 >

Assuming that some attribute weight information is already known and the attribute weights information is shown in the following set:

42 $$ \begin{aligned} {\mathbf{H}} & = \{ {\mathbf{w}} = (w_{1} ,w_{2} ,w_{3} ,w_{4} ,w_{5} )|0.1 \le w_{1} \le 0.2, \, 0.15 \le w_{2} \le 0.25, \\ & \quad \quad w_{2} \le w_{3} \le 0.3,\;w_{4} - w_{2} \ge 0.1,0.1 \le w_{5} \le 0.2\} \\ \end{aligned} $$

We are requested to determine the best 4PL service provider.

The solution steps are as follows:

*Step 1*: Normalize the decision matrix using the following normalization formula:

43 $$ s_{ij} = \left\{ \begin{gathered} x_{ij} ,o_{j} \in BB \hfill \\ x_{ij}^{c} ,o_{j} \in CC \hfill \\ \end{gathered} \right., $$

where $BB$ represents the set of benefit-type indicators and $CC$ represents the set of cost-type indicators.

In this example, only $o_{1}$ belongs to the cost-effectiveness criterion, therefore the normalized SF decision matrix is shown in Table 5.

**Table 5:** Normalized SF decision matrx.

	$o_{1}$	$o_{2}$	$o_{3}$	$o_{4}$	$o_{5}$
$A_{1}$	< 0.1, 0.8, 0.2 >	< 0.6, 0.4, 0.4 >	< 0.5, 0.5, 0.4 >	< 0.6, 0.1, 0.1 >	< 0.6, 0.4, 0.4 >
$A_{2}$	< 0.3, 0.6, 0.4 >	< 0.5, 0.4, 0.4 >	< 0.5, 0.4, 0.4 >	< 0.6, 0.2, 0.1 >	< 0.9, 0.0, 0.1 >
$A_{3}$	< 0.4, 0.3, 0.6 >	< 0.9, 0.1, 0.1 >	< 0.6, 0.4, 0.4 >	< 0.9, 0.1, 0.0 >	< 0.6, 0.4, 0.3 >
$A_{4}$	< 0.3, 0.7, 0.3 >	< 0.3, 0.6, 0.3 >	< 0.4, 0.5, 0.4 >	< 0.6, 0.1, 0.0 >	< 0.6, 0.3, 0.4 >

*Step 2:* Calculate the scores of each alternative solution for each attribute value $x_{ij} = < \mu_{ij} ,\eta_{ij} ,\upsilon_{ij} >$, and obtain the SF score matrix $\user2{S = }(S_{ij} )_{m \times n}$ as shown in Table 6.

**Table 6:** The SF score matrix $\user2{S = }(S_{ij} )_{m \times n}$.

	$o_{1}$	$o_{2}$	$o_{3}$	$o_{4}$	$o_{5}$
$A_{1}$	0.6108	0.3716	0.3516	0.3600	0.3716
$A_{2}$	0.3016	0.2616	0.2616	0.3900	0.8000
$A_{3}$	− 0.0575	0.8100	0.3716	0.8200	0.4338
$A_{4}$	0.4938	0.3638	0.2616	0.3700	0.3016

*Step 3.* Determine the PIS and NIS, as shown in Table 7.

**Table 7:** PIS and NIS.

	$o_{1}$	$o_{2}$	$o_{3}$	$o_{4}$	$o_{5}$
$A^{ * }$	< 0.4, 0.8, 0.6 >	< 0.9, 0.6, 0.4 >	< 0.6, 0.5, 0.4 >	< 0.9, 0.2, 0.1 >	< 0.9, 0.4, 0.9 >
$A^{ - }$	< 0.1, 0.3, 0.2 >	< 0.3, 0.1, 0.1 >	< 0.4, 0.4, 0.4 >	< 0.6, 0.1, 0.0 >	< 0.6, 0.0, 0.1 >

*Step 4.* According to Eqs. (17) and (18), calculate the attribute values of each alternative and the corresponding distances to the PIS and NIS, respectively. The results are shown in Tables 8 and 9.

**Table 8:** The set of distances between each attribute value and the corresponding value of PIS.

	$x_{1}^{ + }$	$x_{2}^{ + }$	$x_{3}^{ + }$	$x_{4}^{ + }$	$x_{5}^{ + }$
$A_{1}$	0.1180	0.1618	0.0581	0.1005	0.4082
$A_{2}$	0.2628	0.1298	0.0390	0.1258	0.1005
$A_{3}$	0.2002	0.3215	0.0431	0.1976	0.1118
$A_{4}$	0.1437	0.2366	0.0821	0.2217	0.1118

**Table 9:** The set of distances between each attribute value and the corresponding value of NIS.

	$x_{1}^{ + }$	$x_{2}^{ + }$	$x_{3}^{ + }$	$x_{4}^{ + }$	$x_{5}^{ + }$
$A_{1}$	0.2155	0.2741	0.0431	0.1976	0.1005
$A_{2}$	0.2002	0.2895	0.0610	0.1826	0.4082
$A_{3}$	0.2628	0.2315	0.0821	0.1005	0.3888
$A_{4}$	0.2215	0.2965	0.0431	0	0.3651

*Step 5.* Calculate the PUVs of the DM for each alternative attribute value $x_{ij}$. Here we set $\theta = 0.88,\xi = 0.20$, and the results are shown in Table 10.

**Table 10:** The PUVs of the DM for each alternative attribute value.

	$o_{1}$	$o_{2}$	$o_{3}$	$o_{4}$	$o_{5}$
$A_{1}$	0.3703	0.3318	0.3034	0.4590	0.7409
$A_{2}$	0.6294	0.4546	0.4038	0.4195	0.4887
$A_{3}$	-0.0632	0.8040	0.4277	0.8145	0.5426
$A_{4}$	0.5544	0.4211	0.2974	0.3611	0.4060

*Step 6.* Based on Eq. (37), establish the following linear programming model:

44 $$ \begin{gathered} \max \;1.4865w_{1} + 2.0056w_{2} + 1.4324w_{3} + 2.0634w_{4} + 2.1610w_{5} \hfill \\ s.t.\;\left\{ \begin{gathered} 0.1 \le w_{1} \le 0.2 \hfill \\ 0.15 \le w_{2} \le 0.25 \hfill \\ w_{2} \le w_{3} \le 0.3 \hfill \\ w_{4} - w_{2} \le 0.1 \hfill \\ 0.1 \le w_{5} \le 0.2 \hfill \\ w_{1} + \cdots + w_{5} = 1 \hfill \\ w_{1} , \cdots ,w_{5} \ge 0 \hfill \\ \end{gathered} \right. \, \hfill \\ \end{gathered} $$

Using Matlab to solve the above optimization model (44), the attribute weights are obtained as follows:

$$ w_{1} = 0.20,w_{2} = 0.25,w_{3} = 0.10,w_{4} = 0.25,w_{5} = 0.20. $$

*Step 7.* Calculate the comprehensive PUVs of each alternative, and we can get

$$ PUV(A_{1} ) = 0.4508,PUV(A_{2} ) = 0.4787,PUV(A_{3} ) = 0.5432,PUV(A_{4} ) = 0.4173. $$

The alternatives are sorted in descending order according to $PUV(A_{i} )$. The sorting result is $A_{3} \succ A_{2} \succ A_{1} \succ A_{4}$, and $A_{3}$ is the best choice. The new assessment method of service providers not only considers the score function but also takes into account the regret-rejoice value of the DM’s alternative selection, which is in line with objective reality.

## Conclusions

The present study was envisaged at the problem of SF MADM with partially known attribute weight. We first constructed a distance formula for Spherical fuzzy sets based on the Hellinger distance of discrete distributions. Through analysis of some examples, we found that the newly proposed distance measure is reasonable and effective. Based on this, we further proposed a Spherical fuzzy MADM method based on regret theory. Our MADM method considered the psychological factors of DMs, which was more in line with reality. In the proposed MADM algorithm, attribute weights are ontained by solving an optimization model, which offered the maximum optimal comprehensive PUV under given weighting information. The new method enriched and developed the weight attribute determination method. According to the values of the comprehensive perceived utility value, the alternatives were ranked. Furthermore, using an illustration of supplier provider evaluation, it was found that the proposed method was effective and feasible.

The method in this paper also has its shortcomings. For instance, we only used numerical examples to demonstrate the advantages of the new distance and the existing distances. However, there is still a lack of comparative studies between decision-making methods based on the new distance of spherical fuzzy sets and other decision-making methods.

Our future work will be to apply the new distance to areas such as image processing and system identification. Furthermore, we will develop the Hellinger distance and the regret theory based MADM to other fuzzy environments, such as T-SF set and q-rung orthopair fuzzy set. We also envisage solving other decision making problems, such as the risk evaluation, system optimization, and material selection using the proposed method.

## Data Availability

All data generated or analysed during this study are included in this published article.
